# Study of the Affinity between the Protein Kinase PKA and Peptide Substrates Derived from Kemptide Using Molecular Dynamics Simulations and MM/GBSA

**DOI:** 10.1371/journal.pone.0109639

**Published:** 2014-10-02

**Authors:** Karel Mena-Ulecia, Ariela Vergara-Jaque, Horacio Poblete, William Tiznado, Julio Caballero

**Affiliations:** 1 Departamento de Ciencias Químicas, Facultad de Ciencias Exactas, Universidad Andres Bello, Santiago de Chile, Chile; 2 Centro de Bioinformática y Simulación Molecular, Facultad de Ingeniería, Universidad de Talca, Talca, Chile; Instituto de Tecnologica Química e Biológica, UNL, Portugal

## Abstract

We have carried out a protocol in computational biochemistry including molecular dynamics (MD) simulations and MM/GBSA free energy calculations on the complex between the protein kinase A (PKA) and the specific peptide substrate Kemptide (LRRASLG). We made the same calculations on other PKA complexes that contain Kemptide derivatives (with mutations of the arginines, and with deletions of N and C-terminal amino acids). We predicted shifts in the free energy changes from the free PKA to PKA-substrate complex (ΔΔG_E→ES_) when Kemptide structure is modified (we consider that the calculated shifts correlate with the experimental shifts of the free energy changes from the free PKA to the transition states (ΔΔG_E→TS_) determined by the catalytic efficiency (k_cat_/K_M_) changes). Our results demonstrate that it is possible to predict the kinetic properties of protein kinases using simple computational biochemistry methods. As an additional benefit, these methods give detailed molecular information that permit the analysis of the atomic forces that contribute to the affinity between protein kinases and their substrates.

## Introduction

The reversible phosphorylation of proteins, catalyzed by protein kinases (PKs), regulates the most important processes in the cell such as the gene transcription and metabolic pathways [1]. On the other hand, aberrant phosphorylation is associated with several disorders. For this reason, PKs have become important targets for rational drug design against cancer, Alzheimer, etc [2], [3]. The phosphoryl-transfer mechanism catalyzed by PKs has been studied in several works using computational chemistry methods [4], [5]; however, no work on computational chemistry studied PKs specificity and selectivity for substrates, which are very important topics since the differential recognition of substrates is essential for the adequate regulation of the cell. The specificity of PKs, referring to discrimination between substrates, arises from the three-dimensional (3D) structure of their active sites, that are sterically and electrostatically complementary to their substrates. Although the overall structures of PKs are very similar, they are also very selective, being capable of discriminating between closely related sequences that contain serine, threonine or tyrosine with different neighbor amino acids.

The catalytic subunit of protein kinase A (PKA) is the best characterized member of the large family of PKs, considering structural, biochemical, and physiological data. In this sense, PKA serves as a paradigm for the whole family [6], [7]. Protein kinase A (PKA) or cAMP-dependent protein kinase (since its activity is dependent on cellular levels of cyclic AMP) is a serine/threonine protein kinase (PK) that regulates glycogen, sugar, and lipid metabolism. The selectivity of PKA is essential for cell integrity. It phosphorylates specific targets in the cell that contain a sequence pattern in the amino acid residues surrounding the phosphorylation site [8]. The optimal recognition motif for PKA is the sequence RRX(S/T)X, with a great importance of arginines at the −3 and −2 positions where position 0 is the primed phosphorylation site [9]. Kemp et al. designed the peptide Kemptide (LRRASLG) with kinetic constants comparable to native protein substrates [10]. They also found that substituting the arginine residues for other residues (alanine, lysine, or histidine), or reducing the chain length in Kemptide, negatively affect the kinetic constants [11]. In a general analysis, authors noted that these changes in Kemptide resulted in less affinities between PKA and substrates. The principal aim of this work is the use of simple computational biochemistry methods to reproduce the effects of changes in Kemptide on the kinetics of PKA-catalyzed phosphorylation. As a result we will create a predictive theoretical model and provide atomistic details about the causes of the differential affinities between PKs and their substrates.

## Materials and Methods

### Preparation of the Initial Structures

We used the initial structure of PKA in complex with a peptide inhibitor (PDB code 1ATP; this crystal also includes ATP and Mn^2+^ atoms) [12]. This structure was modified in VMD software [13]. To construct our system, Mn^2+^ atoms were changed by Mg^2+^. All missing PKA hydrogens were added, and protonation states were assigned for all ionizable residues to their default values at neutral pH. Starting from the peptide inhibitor we build the heptapeptide Kemptide (denoted as WT in this manuscript), which corresponds closely to the peptide sequence of the pig liver pyruvate kinase, a natural PKA substrate [11]. The WT sequence was obtained by mutation of the amino acids of the inhibitor peptide using Mutation plugin of VMD software package [13]. To prepare the mutants, we mutated arginines of the WT sequence inside the transformed PKA model and obtained the models that contain PKA and the peptides LARASLG (R18A), LRAASLG (R19A), LKRASLG (R18K), LRKASLG (R19K), LHRASLG (R18H), and LRHASLG (R19H). We also prepared the models including the shorter chain peptides RRASLG, RASLG, and LRRASL. For the mutants that contain histidine we prepared the models containing neutral histidine with the hydrogen atoms at position δ or ε (H_δ_ and H_ε_), and the models containing the protonated histidine H_p_ (see the next section). All these systems (protein-peptide substrate complexes with ATP and Mg^2+^ ions) were solvated in a octahedron periodic box of TIP3P [14] water molecules with a minimum solute-wall distance of 15 Å. Sodium and chloride counter ions (0.15 M NaCl) were placed on the grid to mimic physiological condition and to guarantee the electric neutrality. This procedure was made using VMD software package [13]. Thereafter, we will perform energy minimization on the models using conjugate gradient method (1000 steps) to reduce any close contacts resulting from the inclusion of new residues.

The experimental values of the variables related to the kinetics of phosphorylation for all the studied peptides are in the Table 1.

**Table 1:** Effect of replacing residues and chain length on kinetics of phosphorylation for the heptapeptide Kemptide (LRRASLG)^a^.

peptide	V_max_ (µmol/min × mg)	K_M_^app^ (µM)	V_max/_K_M_^app^	ΔΔG_E→ES_^exp^ (kcal/mol)	ΔΔG_E→TS_^exp^ (kcal/mol)
LRRA**S**LG	20.2	16.0	1.26250	0.00	0.00
LARA**S**LG	8.7	4900.0	0.00178	3.39	3.89
LRAA**S**LG	5.3	6300.0	0.00084	3.54	4.33
LKRA**S**LG	17.1	1400.0	0.01221	2.65	2.75
LRKA**S**LG	16.9	260.0	0.06500	1.65	1.76
LHRA**S**LG	12.1	415.0	0.02916	1.93	2.23
LRHA**S**LG	6.5	1340.0	0.00485	2.62	3.29
RRA**S**LG	17.9	26.0	0.68846	0.29	0.36
RA**S**LG	10.2	4400.0	0.00232	3.33	3.73
LRRA**S**L	18.1	57.0	0.31754	0.75	0.82

a phosphorilated residue is in bold letter; n means negligible activities. Data from reference [11].

### Special considerations for histidine mutants

**Figure 1:**
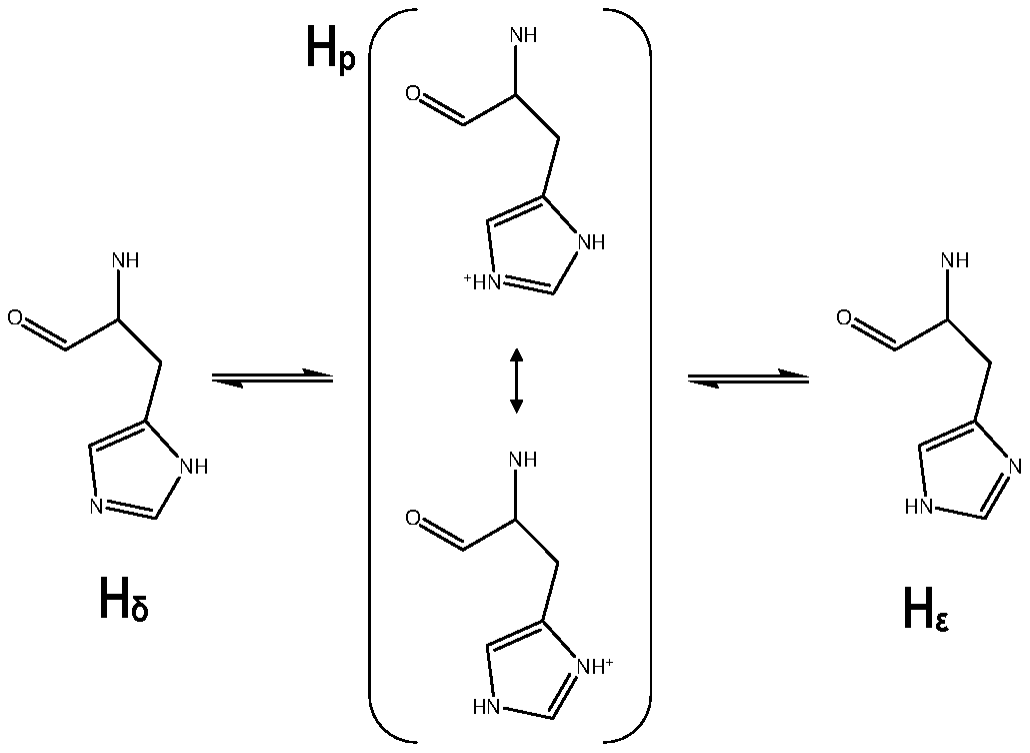
Protonation states of the histidine.

The histidine contains the ionizable imidazole ring at the side chain. The histidine pKa value is approximately 7; therefore, both the acidic and basic forms are present at physiological pH. The acidic form (named H_p_ in this manuscript) has the imidazolium ion, which is a resonance hybrid of two practically equivalent contributing forms (Figure 1). The basic forms (named H_δ_ and H_ε_ in this manuscript) are produced when either of the two ring nitrogens release a proton. At pH values near 7, all three forms, are present in equilibrium, but differences in the molecular environment can shift this equilibrium.

In simulations based on molecular mechanics formalism it is not possible to consider the gain and loss of a proton. The most common practice is the assignment of a single protonation state and using it throughout the MD simulation [15]. Since our objective is the study of the effect of mutated amino acids in the affinity, we did a special treatment for histidines in the mutants R18H and R19H. We constructed three models for each mutant containing H_δ_, H_ε_, and H_p_ and we accomplished MD simulations for each of them. After the analysis of our results, we suggest which are the most important protonation states for histidines at histidine mutants of Kemptide when this amino acid interacts with the residues at PKA.

### Computational biochemistry protocol: Molecular Dynamic simulations and MM-GBSA calculations

MD simulations were carried out using the NAMD software package [16]. Topology and parameters of proteins included in CHARMM27 force field were used [17], [18]. All the systems were subjected to 2.0 ns of equilibration, and then the production was accomplished during 10.0 ns. The equations of motion were integrated with a 1 fs time step. The van der Waals cutoff was set to 12 Å and the temperature was maintained at 298.15 K with the weak coupling algorithm [19]. Long range electrostatic forces were taken into account by means of the Particle Mesh Ewald (PME) approach [20].

**Figure 2:**
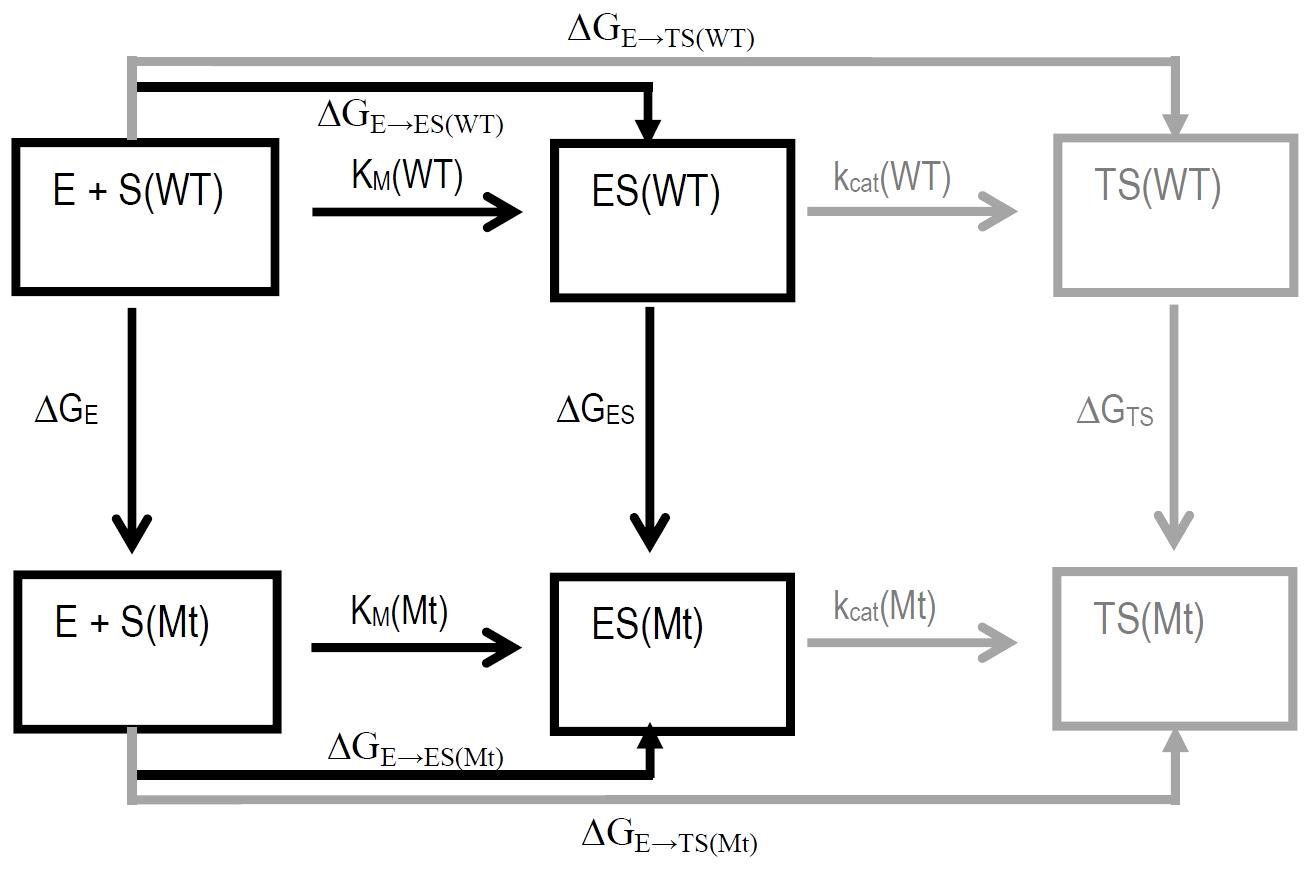
Relationship between free energy changes for the phosphorylation of Kemptide (WT) and mutants (Mt) catalyzed by PKA. E represents the free enzyme, S(i) is the free substrate, ES(i) represents the enzyme-substrate complex, and TS(i) represents the transition state.

We used a computational protocol combining MD simulation and MM-GBSA (Molecular Mechanics-Generalized Born Surface Area) to study PKA-substrate systems. Figure 2 shows the free energy changes associated with the phosphorylation of Kemptide (WT) and mutants (Mt) catalyzed by PKA. As seen in Figure 2, the affinity of the PKA for the substrate is determined by the Gibbs free energy change ΔG_E→ES(i)_ of the reaction system from the reactants (E + S(i)) to the formation of the PKA-substrate complex ES(i), and the catalytic efficiency, that is, k_cat_(i)/K_M_(i), is determined by the Gibbs free energy change ΔG_E→TS_ of the reaction system from the reactants (E + S(i)) to the corresponding rate-determining transition state TS(i).

We executed our study on the PKA-substrate complex, but not the transition state structure. This consideration makes the work much easier because standard MD approaches use classical ‘molecular mechanics’ force fields to simulate a stable molecular system corresponding to a local minimum on the potential energy surface, and the transition state is associated with a maximum potential energy on the potential energy surface. Our study is focused on the study of the effect on ΔG of the amino acids that are close to the serine that is phosphorylated (the free energy shift caused by a structural change in the sequence that contains this serine); therefore, we consider that there is no big change in the shift value when comparing formation of ES(i) with formation of TS(i). Equation 1 represents this consideration for a comparison between the WT and a mutant Mt:
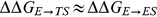
(1)


Considering the thermodynamic cycle in Figure 2 and the Equation (1), it is possible to obtain ΔΔG_E→TS_ using the MD simulation and a free energy calculation of the molecular systems that represent reactants and the PKA-substrate complex.
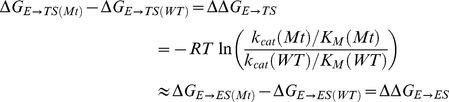
(2)


The ΔG_E→ES_ values were calculated using the MM-GBSA method [21]–[23] by the difference between the PKA, the substrate peptides, and their complexes. The molecular structures were taken from the MD simulations of the complexes. We extracted 900 snapshots from the 10 ns production MD trajectories. The explicit TIP3P water molecules and ions were removed. The free energy was calculated as follows:
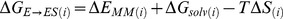
(3)where ΔE_MM(i)_ contains ΔE_internal_ (bond, angle, and dihedral energies), ΔE_elect_ (electrostatic energy), and ΔE_VDW_ (van der Waals energy). ΔG_solv(i)_ is the change of the solvation free energy upon binding, and includes the electrostatic solvation free energy (polar contribution calculated using generalized Born model [24], the dielectric constant of the solvent was set to 78.5), and the nonelectrostatic solvation component (nonpolar contribution estimated by solvent accessible surface area calculated with a probe radius of 1.4 Å). MM-GBSA calculations were achieved in NAMD 2.8 [16]. To reduce computational time, the entropy term TΔS_(i)_ was not calculated since we consider that its contribution is similar for the compared molecular systems. In previous literature, many authors have been reported that the lack of the evaluation of the entropy is not critical for calculating the MM-PBSA or MM-GBSA relative free energies for similar systems [25]–[29].

## Results and Discussion

### Analysis of the Kemptide Arg18 and Arg19 mutants

MD simulations give trajectories that contain structural data of PKA-substrate complexes. Initially, our endeavors were focused on the analysis of the effect of the mutations in the affinity. The dynamics of the interactions between the amino acids of the substrate peptides and residues at the PKA active site were studied. With this information, we give a deeper knowledge about the relevance of the molecular interactions in the complexes between PKA and its substrates.

**Figure 3:**
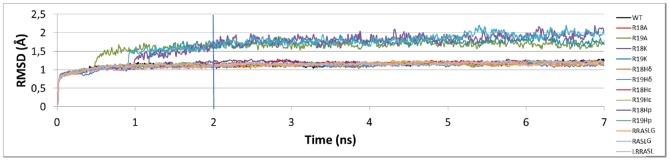
Plots of RMSD values against simulation time corresponding to molecular dynamics of the systems under study. The first 2 ns correspond to equilibration, and the following 5 ns correspond to the first part of the production time.

**Figure 4:**
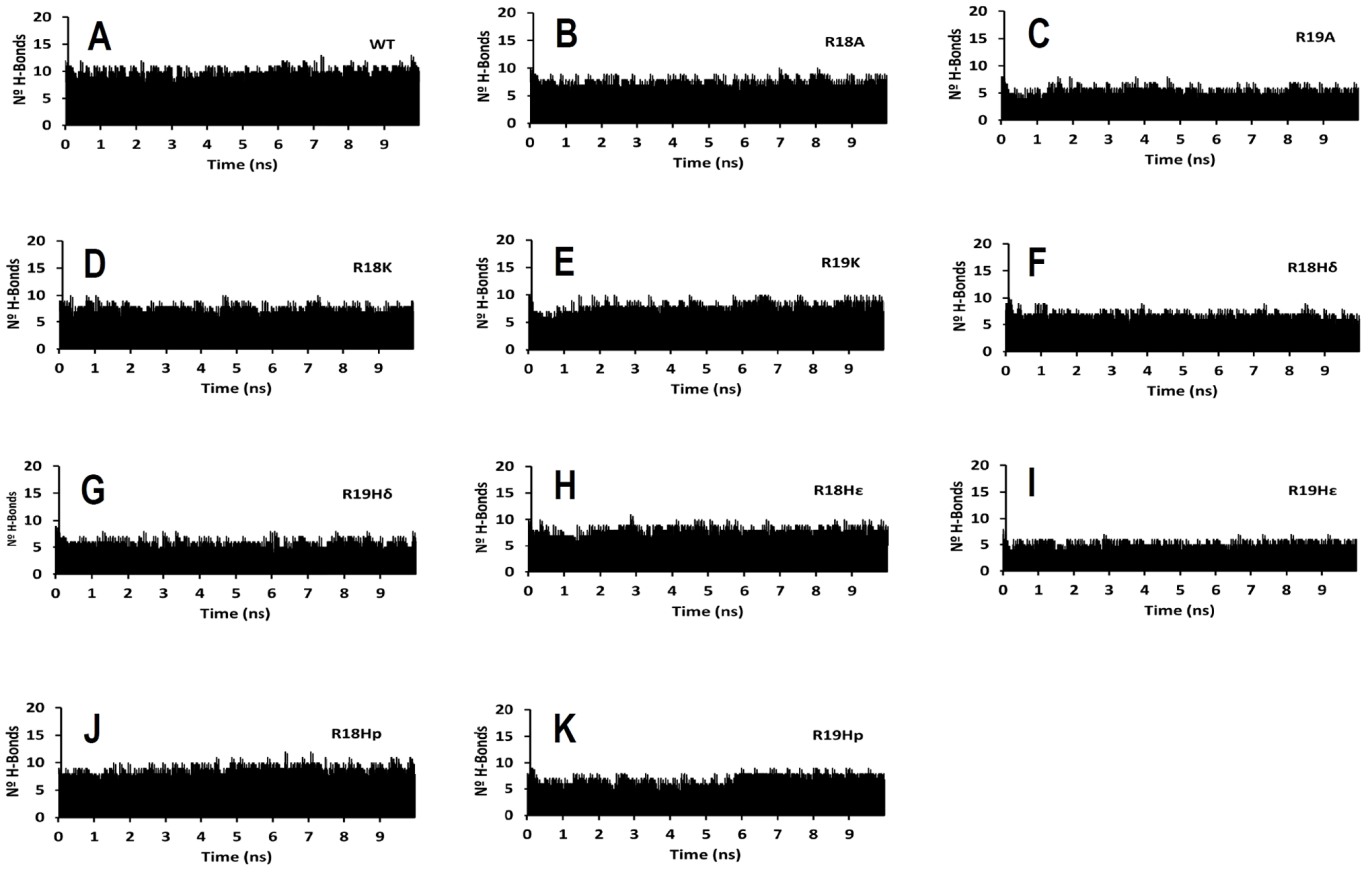
Number of HBs between the PKA and its substrates during 10 ns simulation time: (A) for WT, (B) for R18A, (C) for R19A, (D) for R18K, (E) for R19K, (F) for R18H_δ_, (G) for R19H_δ_, (H) for R18H_ε_, (I) for R19H_ε_, (J) for R18H_p_, (K) and for R19H_p_.

Firstly, we analyzed the stability of the MD trajectories using the rmsd of the positions for all the backbone atoms as a function of simulation time. We observed that rmsd was almost constant after the equilibration process (2.0 ns) for all the systems (Figure 3). We identified the hydrogen bonds (HBs) formed between the substrates and PKA by measuring the donor-aceptor distances during MD simulations. We used a cutoff of 3.5 Å to consider that a donor-aceptor distance is equivalent to an HB. The numbers of HBs between the PKA and its substrates during MD simulation are shown in Figure 4. In general, the network of HB interactions is higher in the complex that contain the WT peptide, and there are less HBs when Arg18 or Arg19 are mutated. In addition, we found less intermolecular HBs when Arg19 is mutated. This result suggests that Arg19 has a higher contribution to the network of HB interactions when comparing with Arg18. Interestingly, the complex that contains the peptide R19A has one of the lower number of HBs. This could explain the lesser binding affinity of the peptide R19A for PKA, and the difference between its affinity and the affinity of the peptide R18A.

**Figure 5:**
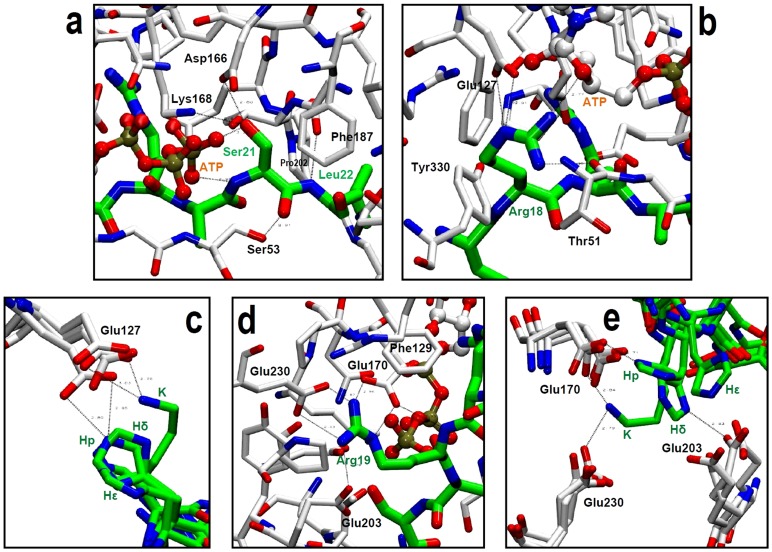
PKA-substrate HB interactions observed in MD simulations for the complexes containing WT and mutant peptides. (A) Interactions of substrate Ser21 and Leu22. (B) Interactions of substrate Arg18. (C) Interactions of Lys and histidines at position 18 of the substrate. (D) Interactions of substrate Arg19. (E) Interactions of Lys and histidines at position 19 of the substrate.

The results of the HBs occupancy during the MD simulations (Tables 2 and 3) allow a comparison of the HB interactions of the residues of Kemptide (WT) and mutants with the amino acids at the substrate PKA binding site. The analysis of these data shows that the side chain hydroxyl of the substrate Ser21 (the residue that is phosphorylated) forms stable HB interactions with the side chain carboxylate of the PKA residue Asp166, the side chain amino group of the PKA residue Lys168, and one of the oxygens of the γ-PO_3_^−^ of the ATP. Meanwhile, the backbone NH group of the Ser21 forms an HB interaction with other of the oxygen atoms of the ATP γ-PO_3_^−^, and the backbone CO group forms an HB interaction with the side chain hydroxyl of the residue Ser53 in the glycine-rich loop of the PKA small lobe (Figure 5a). The backbone NH of the residue at position +1 from the phosphorylated serine (residue Leu22) has a stable HB interaction with the backbone CO of the PKA residue Gly200 in all the studied complexes.

**Table 2:** Hydrogen-Bond occupancies analysis from the results of MD simulations for peptide-PKA interactions for the common groups of the mutants derived from Kemptide (WT).

	WT		R18A		R19A		R18K		R19K		R18H_δ_		R19H_δ_		R18H_ε_		R19H_ε_		R18H_p_		R19H_p_	
Peptide-PKA atoms.^a^	Occupancy(%).^b^	Distance (Å).^c^	Occupancy(%).^b^	Distance (Å).^c^	Occupancy(%).^b^	Distance (Å).^c^	Occupancy(%).^b^	Distance (Å).^c^	Occupancy(%).^b^	Distance (Å).^c^	Occupancy(%).^b^	Distance (Å).^c^	Occupancy(%).^b^	Distance (Å).^c^	Occupancy(%).^b^	Distance (Å).^c^	Occupancy(%).^b^	Distance (Å).^c^	Occupancy(%).^b^	Distance (Å).^c^	Occupancy(%).^b^	Distance (Å).^c^
Arg18-NE - Glu127-OE1	96.10	3.38±0.11			100	2.76±0.14			99.37	2.83±0.18			99.20	2.94±0.19			99.60	2.92±0.17			98.84	2.89±0.19
Arg18-NE - Glu127-OE2	99.00	2.99±0.13			100	3.64±0.26			53.37	3.55±0.29			99.10	3.08±0.19			99.70	3.08±0.17			87.89	3.28±0.27
Arg18-NE - Tyr330-OH	68.00	3.50±0.17			57.14	3.74±0.32			21.68	3.71±0.37			71.96	3.49±0.21			67.27	3.51±0.21			11.05	4.73±1.30
Arg18-NH1 - Thr51-O	99.70	2.97±0.10			100	3.86±0.60			99.79	2.83±0.16			99.50	2.95±0.19			99.30	2.96±0.21			99.16	2.89±0.20
Arg18-NH2 - ATP-O3′	100	2.98±0.13			100	3.13±0.21			99.79	2.99±0.16			100	2.99±0.13			100	3.00±0.13			99.89	3.03±0.16
Arg18-NH2 - Glu127-OE1	39.00	3.28±0.62																				
Arg18-NH2 - Glu127-OE2	34.20	3.04±0.25			100	2.72±0.11			100	2.70±0.11			100	2.79±0.13			100	2.73±0.12			99.89	2.70±0.11
Arg18-NH2 - Thr51-O	19.10	3.18±0.32			100	4.71±0.76			76.32	3.36±0.32			97.31	3.10±0.23			95.31	3.15±0.25			99.16	2.89±0.20
Arg19-N - Glu170-OE2	85.50	2.83±0.11	75.35	3.09±0.18	100*	2.83±0.20	100	2.83±0.12	100*	2.81±0.13	100	2.84±0.12	100*	2.71±0.09	100	2.84±0.12	100*	2.76±0.11	100	2.88±0.14	100*	2.76±0.11
Arg19-NE - Glu170-OE1	75.00	3.50±0.13	72.36	3.50±0.15			60.58	3.55±0.14			63.53	3.54±0.14			69.26	3.53±0.14			68.53	3.53±0.14		
Arg19-NE - Glu170-OE2	98.30	2.73±0.09	98.70	2.71±0.09			100	2.70±0.08			100	2.71±0.09			100	2.71±0.08			100	2.79±0.10		
Arg19-NH1 - Glu203-OE2	87.10	2.81±0.21	95.31	2.77±0.26			90.62	2.90±0.62			24.28	4.17±0.84			89.22	2.99±0.67			65.47	3.36±0.87		
Arg19-NH1 - Glu230-OE1	88.00	2.76±0.12	82.24	2.79±0.13			100	2.81±0.14			100	2.82±0.15			99.90	2.79±0.14			100	2.74±0.11		
Arg19-NH1 - Glu230-OE2	79.80	3.42±0.23	85.23	3.34±0.25			77.94	3.40±0.26			76.68	3.37±0.28			80.54	3.39±0.25			89.26	3.53±0.14		
Arg19-NH2 - Glu170-OE1	97.20	2.74±0.10	97.90	2.75±0.10			100	2.76±0.11			100	2.75±0.10			100	2.74±0.10			100	2.73±0.10		
Arg19-NH2 - Glu170-OE2	89.20	3.41±0.15	91.52	3.38±0.15			96.51	3.33±0.14			91.66	3.38±0.15			91.22	3.39±0.15			82.21	3.45±0.15		
Arg19-NH2 - Glu230-OE1	98.00	3.19±0.12	15.57	3.12±0.19			99.4	3.08±0.19			99.25	3.07±0.20			98.70	3.13±0.20			97.26	3.25±0.20		
Arg19-NH2 - Glu230-OE2	96.80	3.07±0.10	28.74	3.30±0.31			90.12	3.22±0.28			85.45	3.26±0.30			94.41	3.12±0.27			97.26	2.98±0.24		
Ser21-N - ATP-O1G	98.80	3.11±0.19	64.17	3.37±0.45	100	4.29±1.10	100	2.96±0.15	99.47	3.03±0.18	99.79	3.00±0.17	96.11	3.20±0.20	99.90	3.00±0.16	99.90	3.01±0.16	98.42	3.05±0.21	99.47	2.99±0.17
Ser21-O - Ser53-OG	66.40	2.77±0.14	28.64	3.29±0.48	100	3.74±0.79	96.21	2.87±0.25	100	2.72±0.11	100	2.79±0.15	99.90	2.78±0.15	99.90	2.73±0.13	99.90	2.78±0.14	100	2.74±0.16	98.74	2.82±0.23
Ser21-OG - Asp166-OD1	100	2.72±0.17	9.08	3.16±0.54	100	4.29±0.86	99.50	3.00±0.21	87.26	3.28±0.29	99.68	2.93±0.19	99.90	2.89±0.21	99.90	2.77±0.19	99.70	2.99±0.19	79.89	3.38±0.28	80.95	3.40±0.25
Ser21-OG - ATP-O3G	66.00	3.44±0.33	82.93	3.12±0.40	95.92	3.80±0.72	95.71	2.95±0.29	94.42	2.92±0.31	95.29	2.96±0.30	91.52	3.06±0.33	61.88	3.42±0.40	98.90	2.89±0.24	98.53	2.89±0.23	98.00	2.83±0.22
Ser21-OG - Lys168-NZ	96.20	2.87±0.14	85.13	2.90±0.18	100	2.91±0.16	99.30	2.93±0.18	100	2.87±0.13	99.68	2.91±0.16	100	2.86±0.13	99.90	2.86±0.14	99.60	2.89±0.15	100	2.84±0.12	99.89	2.84±0.12
Leu22-N - Gly200-O	63.70	2.93±0.17	100	2.96±0.15	100	2.91±0.16	99.60	2.97±0.17	99.79	2.87±0.15	99.79	2.97±0.16	99.70	2.95±0.17	100		99.80	3.00±0.16	99.16	2.97±0.18	98.42	2.95±0.21

a Peptide-PKA heavy atoms that form a HB (Notations of atoms refer to the name of atoms from the CHARMM force field). The listed donor and acceptor pairs satisfy the criteria for the HB over 50% of time during the whole MD simulation (distance between heavy atoms <3.5 Å, values higher than 3.5 are included only for comparison).

b The occupancies reflect the % of the time that the HB exists with respect to the whole time,

* indicates that the backbone N atom of Arg19 really represents the backbone N atom of the mutated amino acid.

c The averaged distance ± standard deviation between hydrogen-acceptor and hydrogen-donor heavy atoms during the time that the HB is formed.

**Table 3:** Hydrogen-Bond occupancies analysis from the results of MD simulations for the peptide-PKA interactions between mutated amino acids.

Mutant	Peptide-PKA atoms.^a^	Occupancy(%)^b^	Distance (Å)^c^
R18K	Lys18-NZ - Glu127-OE1	96.31	2.88±0.28
	Lys18-NZ - Glu127-OE2	95.41	2.88±0.29
R19K	Lys19-NZ - Glu170-OE1	92.42	2.99±0.46
	Lys19-NZ - Glu170-OE2	92.95	2.95±0.39
	Lys19-NZ - Glu230-OE1	80.42	3.35±0.50
R18H_δ_	Hsd18-ND1 - Glu127-OE1	34.01	3.76±0.42
	Hsd18-ND1 - Glu127-OE2	3.85	4.46±0.60
R19H_δ_	Hsd19-ND1 - Glu203-OE1	99.50	2.90±0.17
	Hsd19-ND1 - Glu203-OE2	65.47	3.49±0.26
R18H_ε_	Hse18-NE2 - Glu127-OE1	76.45	3.28±0.65
	Hse18-NE2 - Glu127-OE2	75.35	3.46±0.81
R19H_ε_	Hse19-NE2 - Glu203-OE1	37.13	3.88±0.65
	Hse19-NE2 - Glu203-OE2	42.71	3.79±0.71
R18H_p_	Hsp18-NE2 - Glu127-OE1	98.21	2.78±0.24
	Hsp18-NE2 - Glu127-OE2	93.37	3.10±0.30
R19H_p_	Hsp19-ND1 - Glu170-OE2	100	2.68±0.10
	Hsp19-NE2 - Glu203-OE1	20.21	4.25±0.76

a Peptide-PKA heavy atoms that form a HB (Notations of atoms refer to the name of atoms from the CHARMM force field). The listed donor and acceptor pairs satisfy the criteria for the HB over 50% of time during the whole MD simulation (distance between heavy atoms <3.5 Å, values higher than 3.5 are included only for comparison).

b The occupancies reflect the % of the time that the HB exists with respect to the whole time.

c The averaged distance ± standard deviation between hydrogen-acceptor and hydrogen-donor heavy atoms during the time that the HB is formed.

The guanidine side chain group of the residue Arg18 of the peptide substrates (the residue at position -3 from the phosphorylated serine) has stable HB interactions with the side chain carboxylate group of the PKA residue Glu127, with the backbone CO of the residue Thr51 in the glycine-rich loop of the PKA small lobe, and with the hydroxyl at the position 3 of the D-ribofuranose ATP moiety (Figure 5b). The analysis of the occupancies also shows that the side chain of Arg18 can form non-stable HB interactions with the side chain hydroxyl of the PKA residue Tyr330. Table 3 shows that the side chain groups of the mutated residues Lys and the protonated histidine at position 18 of the WT substrate (in mutants R18K and R18H_p_) also form HB interactions with the side chain carboxylate group of the PKA residue Glu127 (Figure 5c). These interactions are weaker for histidine ε (mutant R18H_ε_), and even weaker for histidine δ (mutant R18H_δ_). This interaction is not present in the mutant R18A.

The guanidine side chain group of the residue Arg19 of the peptide substrates (the residue at position -2 from the phosphorylated serine) has stable HB interactions with the side chain carboxylate groups of the PKA residues Glu170, Glu203, and Glu230 (Table 2, Figure 5d). At the same time, the backbone NH of Arg19 forms an HB interaction with the side chain carboxylate of the PKA residue Glu170; this interaction is conserved when Arg19 is changed by other amino acids in all the studied mutants.

Table 3 shows that the side chain groups of the mutated residues Lys and the protonated histidine at position 19 of the WT substrate (in mutants R19K and R19H_p_) also form HB interactions with the side chain carboxylate group of the PKA residue Glu170, while the mutated residue histidine δ at the same position 19 (in mutant R19H_δ_) also forms an HB interaction with the side chain carboxylate group of the PKA residue Glu203 (Figure 5e). The histidine ε at this position has no stable HB interactions with PKA residues.

It is interesting to note, after the analysis of occupancies of the HB interactions, that the mutants that contain alanine at positions 18 and 19 of the substrate (R18A and R19A) do not contain groups able to form HBs with PKA; therefore, these mutants cannot form the interactions represented in the Figures 5b and d, respectively. It is also noteworthy the presence of a more stable and bigger HB network established by Arg19 with the residues at PKA, when comparing with the network established by Arg18. The substitution of the arginine at this position for lysine or histidine retains only a part of the original interactions; therefore, arginine looks optimal in the pocket that contains substrate residues at position 19, where three glumatates capture the guanidine group with a great molecular force forming a very stable framework.

The hydrophobic interactions were analyzed by accounting for stable C-C distances <4.5 Å. We observed that the PKA residue Phe187 in the activation segment has a hydrophobic interaction with the substrate residue Ser21 and the PKA residue Pro202 has a hydrophobic interaction with the substrate residue Leu22 for all the complexes (Figure 5a). In previous results, Li et al. [30] demonstrated the positive cooperativity of ATP and the PKA residues Thr51, Glu170, and Phe187 using a series of MD simulations and free energy calculations. They also identified the interactions mentioned here for the complex between PKA and Kemptide.

The MM-GBSA free energy calculations were employed to get quantitative estimates for the binding free energies of the WT and the mutants inside the PKA active site. We calculated ΔΔG_E→ES_ values and compared them with experimental ΔΔG_E→TS_ values. There is a high correlation between experimental ΔΔG_E→TS_ and ΔΔG_E→ES_ values (R^2^ = 0.988 including all the peptides in Table 1) that confirm that our data satisfy the Equation (1). Therefore, the calculation of ΔΔG_E→ES_ instead of ΔΔG_E→TS_ is pertinent to get thermodynamic information of PKA-substrate systems. In other words, it is not necessary to describe the transition state; this is important because it is possible the use of a simple method such as MM-GBSA instead of other more complex method accounting for the quantum mechanical effects during transition state formation.

**Figure 6:**
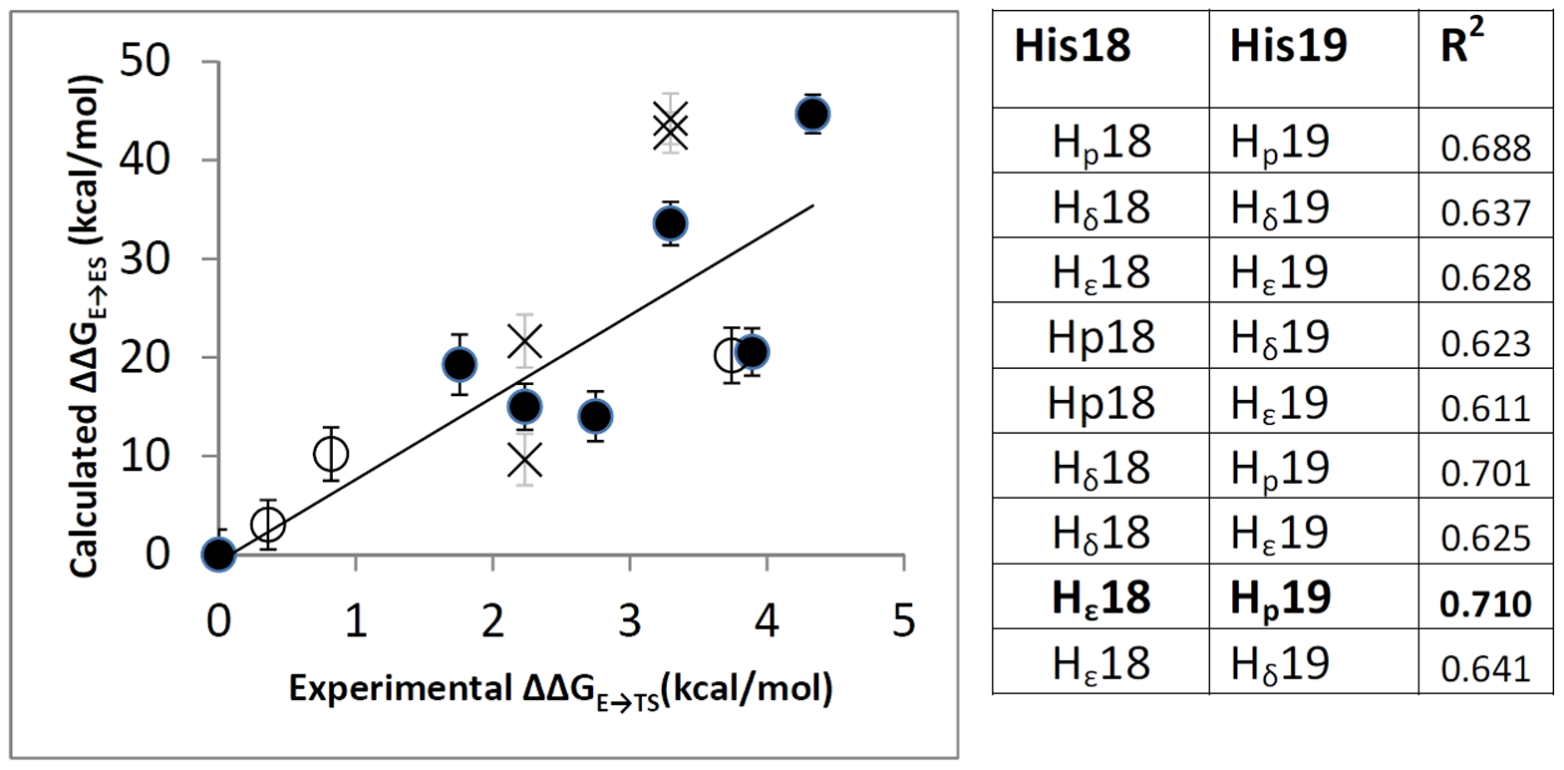
Relationship between experimental ΔΔG_E→TS_ and calculated ΔΔG_E→ES_ values using MM-GBSA. (left) Plot of the relationship: mutants included in the best correlation are represented with close circles, mutants that include a histidine that was not selected in the best correlation are represented with a cross, peptides with smaller chain are represented with open circles. (right) Different R^2^ values when different pairs of histidine mutants are included in the correlation, the best correlation is in bold letter.

We have to consider that three classical histidines (H_δ_, H_ε_, and H_p_) were used to model R18H and R19H mutants. To establish the correlation between experimental and theoretical ΔΔG values we selected only one of them for each mutant; although, a thorough chemical analysis indicates that all of them should contribute to the real system. The better contribution to the correlation of one of the histidines should indicate which is the preponderant protonation state of histidine at positions 18 and 19 of the substrate when they interact with PKA active site. We evaluated the correlations for the mutants when different pairs of classical histidines are incorporated. It is noteworthy that there is a linear correlation (R^2^>0.6 when different pairs of histidines are included, Figure 6) between the predicted ΔΔG_E→ES_ and the experimental ΔΔG_E→TS_. These correlations indicate that the parameters applied to calculate free energy changes are feasible and reliable. The best correlation with a coefficient R^2^ = 0.710 was found when H_ε_ is at position 18 and H_p_ is at position 19. The plot when mutants R18H_ε_ and R19H_p_ are included is represented in Figure 6. The effect in R^2^ of changing protonated states at positions 18 and 19 is analyzed. It is noteworthy that the presence of H_p_ at position 19 of the substrate gives higher R^2^ values; when H_p_ at position 19 is changed by H_δ_ or H_ε_ then R^2^ decreases. This analysis suggests that histidine remains in a single positive ionization state at position 19 in the mutant R19H to establish electrostatic interactions inside the pocket formed by the PKA residues Glu170, Glu203, and Glu230 (Figure 5e). On the other hand, the presence of different histidines at position 18 does not have a clear effect on the correlation coefficient R^2^, which suggests that protonated and unprotonated states are allowed at position 18. The electrostatic interactions between the substrate and the PKA pocket in this zone are less strong when comparing with the interactions with the pocket at position 19.

Mutations try to mimic the original PKA-substrate interactions of arginines when Kemptide is present. Histidines in mutants R18H and R19H should be protonated to mimic the original arginines, but the histidine 18 does not need to be protonated according to our results. This result suggests that a protonated residue is more important at position 19 (position −2 from phosphorylated serine); therefore, the arginine at this position is more important for PKA-substrate affinity. The presence of a protonated residue at the position 18 (position −3 from phosphorylated serine) is less important for PKA-substrate affinity.

Since the affinity trend is successfully reproduced, we can analyze the free energy components such as van der Waals (VDW), electrostatic, and solvation contributions to give detailed molecular information about the studied systems. The results of the predicted ΔG_E→ES_ values and ΔE_vdw_, ΔE_ele_, and ΔG_solv_ components for the complexes were summarized in Table 4. To get a better view on which energy terms have more impact on the change in PKA-substrate affinity, these individual energy components were carefully compared. From Table 4, it can be seen that ΔE_vdw_ has the major favorable contribution to the total free energy, but there is no big difference among the ΔE_vdw_ value for different PKA-substrate complexes. In this sense, the VDW energy term is not primarily responsible for differentiating the binding affinity of Kemptide and its mutants. The solvation term also has the same effect in all the PKA-substrate complexes with a favorable contribution to the global free energy. These terms neither influence the difference between the calculated ΔΔG_E→ES_ values.

**Table 4:** Predicted relative MM-GBSA free energies (kcal/mol) and individual energy terms of the complexes between mutant peptides derived from Kemptide and PKA.

	ΔG_E→ES_^calc^ a (kcal/mol)	ΔE_VDW_ (kcal/mol)	ΔE_ele_ (kcal/mol)	ΔG_solv_ (kcal/mol)	ΔΔG_E→ES_^calc^ (kcal/mol)
WT	−73.34±2.54	−38.83±2.26	−27.30±2.25	−7.22±1.95	0.00±2.54
R18A	−52.79±2.39	−34.34±2.45	−12.16±2.21	−6.29±2.03	20.55±2.39
R19A	−28.66±1.94	−34.20±2.32	11.37±2.16	−5.83±2.06	44.68±1.94
R18K	−59.32±2.52	−35.46±2.07	−16.86±2.16	−5.91±2.22	14.02±2.52
R19K	−54.07±3.05	−41.52±2.16	−5.38±2.23	−7.16±1.99	19.27±3.05
R18H_δ_	−51.69±2.69	−40.52±2.46	−4.27±2.53	−6.90±2.05	21.65±2.69
R19H_δ_	−30.54±2.04	−39.29±2.15	15.67±2.19	−6.93±2.01	42.79±2.04
R18H_ε_	−58.33±2.35	−39.01±2.55	−12.05±2.21	−7.28±1.95	15.00±2.35
R19H_ε_	−29.14±2.57	−38.26±2.44	16.48±2.23	−7.36±1.99	44.20±2.57
R18H_p_	−63.70±2.61	−36.47±2.66	−20.49±2.33	−6.74±2.01	9.63±2.61
R19H_p_	−39.76±2.19	−36.62±2.15	3.49±2.21	−6.63±2.10	33.58±2.19

a Calculated binding energies do not include entropy term.

The most important term which dictates the difference in the binding affinity is ΔE_ele_. The better binding of Kemptide gains over 6.81 kcal/mol of ΔE_ele_ value compared with the next favourable value for a mutant (R18H_p_). In this sense, ΔE_ele_ is the key factor for the more favorable free energy value for Kemptide. This is in agreement with the previous analysis of the importance of HB contributions. It is noteworthy that the ΔE_ele_ value opposes affinity for the mutant models that contain the histidine residues H_δ_, H_ε_, and H_p_ at position 19.

### Analysis of the shorter chain peptides derived from Kemptide

MD simulations of the PKA-substrate systems including Kemptide-derived shorter chain peptides have stable rmsd values after the equilibration process (2.0 ns). After this time step, the effect of the removal of residues in PKA-substrate affinity was examined. The occupancies of the HBs formed between the substrates and PKA were identified by measuring the donor-aceptor distances during MD simulations (cutoff of 3.5 Å was used to consider that a donor-aceptor distance is equivalent to an HB; Table 5). The analysis of Table 5 shows that the interactions described above between the substrate residues Arg18, Arg19, Ser21, and Leu22 and the PKA residues and ATP are conserved in shorter chain peptides when the complexes are formed.

**Table 5:** Hydrogen-Bond occupancies analysis from the results of MD simulations for the peptide-PKA interactions for shorter chain peptides derived from Kemptide (WT).

	WT		RRASLG		RASLG		LRRASL	
Peptide-PKA atoms.^a^	Occupancy(%).^b^	Distance (Å).^c^	Occupancy(%).^b^	Distance (Å).^c^	Occupancy(%).^b^	Distance (Å).^c^	Occupancy(%).^b^	Distance (Å).^c^
Arg18-NE - Glu127-OE1	96.10	3.38±0.11	100	2.87±0.15			99.60	2.88±0.17
Arg18-NE - Glu127-OE2	99.00	2.99±0.13	97.70	3.13±0.20			96.01	3.08±0.22
Arg18-NE - Tyr330-OH	68.00	3.50±0.17	67.96	3.51±0.20			69.96	3.50±0.20
Arg18-NH1 - Thr51-O	99.70	2.97±0.10	99.60	2.94±0.19			95.31	3.07±0.26
Arg18-NH2 - ATP-O3′	100	2.98±0.13	99.90	3.00±0.14			100	3.00±0.14
Arg18-NH2 - Glu127-OE1	39.00	3.28±0.62	22.55	3.78±0.27			18.96	3.85±0.31
Arg18-NH2 - Glu127-OE2	34.20	3.04±0.25	100	2.71±0.12			100	2.76±0.14
Arg18-NH2 - Thr51-O	19.10	3.18±0.32	93.51	3.16±0.26			87.62	3.26±0.51
Arg19-N - Glu170-OE2	85.50	2.83±0.11	100	2.77±0.10	100	2.66±0.08	100	2.80±0.11
Arg19-NE - Glu170-OE1	75.00	3.50±0.13	81.54	3.46±0.14	75.35	3.51±0.14	67.27	3.53±0.13
Arg19-NE - Glu170-OE2	98.30	2.73±0.09	100	2.75±0.09	100	2.72±0.09	100	2.71±0.08
Arg19-NH1 - Glu203-OE2	87.10	2.81±0.21	97.21	2.81±0.27	98.40	2.81±0.23	87.33	3.00±0.70
Arg19-NH1 - Glu230-OE1	88.00	2.76±0.12	100	2.78±0.12	99.90	2.82±0.16	100	2.78±0.03
Arg19-NH1 - Glu230-OE2	79.80	3.42±0.23	82.04	3.39±0.23	82.04	3.37±0.25	78.44	3.40±0.25
Arg19-NH2 - Glu170-OE1	97.20	2.74±0.10	100	2.74±0.10	100	2.73±0.10	100	2.75±0.11
Arg19-NH2 - Glu170-OE2	89.20	3.41±0.15	85.73	3.43±0.15	81.64	3.47±0.15	93.71	3.37±0.14
Arg19-NH2 - Glu230-OE1	98.00	3.19±0.12	98.90	3.15±0.19	99.40	3.10±0.20	99.60	3.10±0.19
Arg19-NH2 - Glu230-OE2	96.80	3.07±0.10	92.91	3.14±0.28	94.01	3.11±0.27	91.02	3.19±0.27
Ser21-N - ATP-O1G	98.80	3.11±0.19	22.26	2.96±0.15	99.70	2.98±0.17	95.31	2.98±0.16
Ser21-O - Ser53-OG	66.40	2.77±0.14	99.50	2.74±0.15	97.50	2.89±0.24	94.31	3.03±0.30
Ser21-OG - Asp166-OD1	100	2.72±0.17	99.60	2.78±0.21	99.60	2.95±0.22	98.80	3.06±0.21
Ser21-OG - ATP-O3G	66.00	3.44±0.33	63.67	5.58±0.35	90.62	3.05±0.34	98.20	2.86±0.23
Ser21-OG - Lys168-NZ	96.20	2.87±0.14	95.41	2.87±0.13	89.92	2.89±0.15	89.02	2.98±0.20
Leu22-N - Gly200-O	63.70	2.93±0.17	99.90	2.91±0.16	99.90	2.94±0.15	99.50	3.05±0.19

a Peptide-PKA heavy atoms that form a HB (Notations of atoms refer to the name of atoms from the CHARMM force field). The listed donor and acceptor pairs satisfy the criteria for the HB over 50% of time during the whole MD simulation (distance between heavy atoms <3.5 Å, values higher than 3.5 are included only for comparison).

b The occupancies reflect the % of the time that the HB exists with respect to the whole time.

c The averaged distance ± standard deviation between hydrogen-acceptor and hydrogen-donor heavy atoms during the time that the HB is formed.

In general, the analysis of the models allows explaining the experimental thermodynamic data, but we also accomplished MM-GBSA free energy calculations to get quantitative estimates for the binding free energies of the shorter chain peptides inside the PKA active site (Table 6). We observed that the lack of the substrate residues Leu17 (in the peptide RRASLG) and Gly23 (in the peptide LRRASL) does not lead to a lack of important PKA-substrate interactions, which is reflected in small experimental and calculated ΔΔG values (Tables 1 and 6). On the other hand, the lack of the substrate residue Arg18 (in the peptide RASLG) leads to the lack of the interactions represented in the Figure 5b, which has a great influence in experimental ΔΔG_E→ES_ and ΔΔG_E→TS_ values (Table 2). This effect was perceived in the theoretical calculation (calculated ΔΔG_E→ES_  = 20.20 kcal/mol).

**Table 6:** Predicted relative MM-GBSA free energies (kcal/mol) and individual energy terms of the complexes between shorter chain peptides derived from Kemptide and PKA.

	ΔG_E→ES_^calc^ a (kcal/mol)	ΔE_VDW_ (kcal/mol)	ΔE_ele_ (kcal/mol)	ΔG_solv_ (kcal/mol)	ΔΔG_E→ES_^calc^ (kcal/mol)
WT	−73.34±2.54	−38.83±2.26	−27.30±2.25	−7.22±1.95	0.00±2.54
RRASLG	−70.30±2.63	−35.71±2.61	−27.08±2.74	−7.52±2.10	3.04±2.63
RASLG	−53.14±2.35	−25.58±2.28	−21.55±2.41	−6.02±2.12	20.20±2.35
LRRASL	−63.13±2.67	−37.11±2.40	−17.63±2.63	−8.40±2.03	10.21±2.67

a Calculated binding energies do not include entropy term.

We analyzed the VDW, electrostatic, and solvation MM-GBSA contributions for the shorter chain peptides. The results of the predicted ΔG_E→ES_ values and ΔE_vdw_, ΔE_ele_, and ΔG_solv_ components for these complexes are summarized in Table 6. To get a better view on which energy terms have more impact on the change in PKA-substrate affinity, these individual energy components were carefully compared. As in the mutants, it can be seen that ΔE_vdw_ has the major favorable contribution to the total free energy, but the contribution is lesser for the systems containing the shorter peptide RASLG. In this sense, the VDW energy term is responsible for differentiating the binding affinity of different chain length substrates. On the other hand, the solvation term has the same effect in all the PKA-substrate complexes with a favorable contribution to the global free energy.

The term ΔE_ele_ has differences for the shorter chain peptides. The lack of the substrate residue Leu17 in the peptide RRASLG has no effect on ΔE_ele_, but the lack of Arg18 in the peptide RASLG harms the electrostatic interactions. Curiously, the electrostatic contribution for the complex that contains the peptide LRRASL is lower than expected (considering that this peptide contains both arginines at positions 18 and 19). We attribute this to the presence of the C-terminal carboxylate in the substrate residue Leu22; the pocket in PKA where Leu22 is located is very hydrophobic, and charged groups have non favorable interactions.

When the calculated ΔΔG_E→ES_ values are inserted in the plot represented in the Figure 6 (open circles), the correlation coefficient R^2^ = 0.714 (this is the correlation coefficient where all the PKA-substrate systems studied in this work are considered). In this sense, we can conclude that our calculations are able to reproduce the trend of the experimental ΔΔG_E→TS_ values, and we can propose the MM-GBSA model as a linear predictor.

### Contribution of computational biochemistry to the study of PKs specificity and selectivity for substrates

Proteins play a main role on the cell functionality, and a very big part of the research today is concerned with the understanding of the interactions of such molecular components. The system biology and proteomics consider the study of the proteins and their functions, which is important in areas such as drug discovery, and understanding of the cell functionalities and the underlying causes behind diseases. Computational biochemistry methods can predict structural and functional details of molecular components. There are many reports involving PK-substrate systems, that can be studied using these methods.

High specificity and catalytic efficiency are the most important characteristics of PKs. Specificity arises from the 3D structure of the PK active site, which is able to include ATP and large substrates (other proteins), and is complementary to the transition state of the reaction. The substrate must fit perfectly within the large PK active site, which can establish many interactions with the phosphorylated residue and the nearest-neighbor residues in the sequence. Small changes in the surrounding amino acids in the sequence can dramatically affect the enzyme action. We demonstrated that our computational biochemistry protocol was able to capture the effect of the structural changes in the PK-substrate affinity for the case of the complexes between PKA and Kemptide derivatives. Despite Kemptide was reported over thirty years ago, the study of the interactions between PKs and their substrates is still a subject of study at present.

We would like to cite in this section other cases of PK-substrate systems, where small changes in the substrate sequence affect the PK catalytic action. We think that a computational protocol similar to our protocol presented here could contribute to explain the experimental findings exposed in the following.

Earlier studies show that several PKs can recognize similar phosphorylation motifs, but exhibit different steady-state kinetics for the same substrate. For instance, Thomas et al. [31] found that bovine lung cGMP-binding cGMP-specific phosphodiesterase (cG-BPDE) is a potent and relatively specific substrate for PKG (cGMP-dependent PK) as compared to PKA. Colbran et al. [32] found that the synthetic peptide RKISASEFDRPLR (BPDEtide), which has the sequence surrounding the phosphorylation site in cG-BPDE, retained the PKG/PKA specificity demonstrated by native cG-BPDE. They also studied peptide analogs of BPDEtide to determine the contribution of specific residues to PKG or PKA substrate specificity. They reported different substitutions that did not reduce PKG/PKA specificity. However, they found that a truncated BPDEtide (RKISASE) served equally well as substrate for both PKs, and a restoration of the phenylalanine, to yield RKISASEF, reproduced the original PKG/PKA specificity.

In other work, Glass and Krebs [33] evaluated the phosphorylation of histone H2B mediated by PKA and PKG. They used as substrates histone H2B, RKRS_32_RKE and RKES_36_YSV, corresponding to the amino acid sequences around Ser32 and Ser36 in histone H2B, and the undecapeptide RKRS_32_RKES_36_YSV containing both serines. They found that PKA preferentially phosphorylated the heptapeptide containing Ser36, rather than that corresponding to Ser32, mainly on the basis of a higher V_max_ for the former substrate. Interestingly, they found that the undecapeptide containing both Ser32 and Ser36 was phosphorylated by PKA with kinetic constants that were identical with those reported for the PKA phosphorylation of Kemptide. On the other hand, based on a lower K_M_, the peptide containing Ser32 is a better phosphate acceptor for PKG than for PKA.

In general, the above mentioned examples show that sequence manipulation can lead to a change in specificity for different PKs. It is possible to create atomistic models of PKA and PKG forming complexes with the above mentioned peptide substrates and develop a protocol of MD and MM/GBSA to help in the elucidation of the interactions that are responsible of the results exposed. In general, the vast information about PK structures allows to create reliable models of the substrates and PKs that are not crystallized to proportionate novel ideas about the structure and function of PK-substrate complexes.

Other examples show the possible applicability of the protocol reported here in the prediction of phosphorylation sites. There are reports on the PK actions on protein substrates that cause some specific response associated with any physiological cellular change. For instance, a direct phosphorylation of Ca^2+^ channel protein by PKA enhances the voltage-gated L-type calcium current by isoproterenol [34]. PKA also increases potassium current when it is stimulated in cardiac ventricular myocytes with a membrane-soluble cAMP analog, indicating that PKs also can regulate K^+^ channels, by phosphorylating some exposed amino acid residues of these proteins [35].

In a more recent report, Michard et al. [36] investigated the modulation of AKT2 (K^+^ channel subunit encoded by the genome of the plant Arabidopsis thaliana) current by effectors of phosphatases and PKs. The authors demonstrated that transitions between AKT2 gating modes 1 and 2 involve phosphorylation/dephosphorylation. They observed that dephosphorylation can result in AKT2 channel silencing; and phosphorylation by a PKA-like PK in the plant favors both recruitment of silenced AKT2 channels and transition from gating mode 1 to gating mode 2. They identified two PKA phosphorylation sites (Ser210 and Ser329) in the region of the pore inner mouth of AKT2. These sites are conserved in AKT2-related channels cloned from other plant species. The PK regulation of the dual gating mode of AKT2 has important consequences in plants since AKT2 is mainly expressed in the mesophyll and phloem tissues [37], [38].

For the application of a computation biochemistry protocol in this example, it is necessary the construction of a 3D model of the PKA-like PK of Arabidopsis and the peptides which has the sequence surrounding the phosphorylation sites Ser210 and Ser329 in AKT2. With the protocol application, it could be possible to evaluate which AKT2 phosphorylation sites are more likely to be identified by the PKA-like PK of Arabidopsis. In addition, these models could help in the design of mutagenesis experiments to study the molecular aspects of AKT2 regulation by phosphorylation, and the possible physiological meaning of such regulation in the plant context.

Our current endeavors are oriented to the application of the computational biochemistry protocol reported here to study the exposed cases.

## Conclusions

The understanding of the basis of PK-substrate specificity is essential for the identification of physiologically important substrates and also to manipulate the metabolic pathway with therapeutic purposes. In the present work, we report a computational biochemistry protocol that gives a theoretical model able to predict the differential affinities of PKA for the classic substrate Kemptide and some derivatives including mutants and short length derivatives.

In summary, the protocol consists of two steps: first, molecular structures representing the complexes in solvent media were subjected to MD simulations, and second, a binding affinity between PKA and substrate was predicted using MM-GBSA free energy calculations. A comparison between the calculated ΔΔG values and experimental ones indicates that the model was able to describe differential affinities. As a result, the trend of the calculated affinities for the studied PKA peptide substrates was highly related to the trend of their experimental catalytic efficiencies (R^2^ = 0.714). In this sense, our model demonstrates that it is possible to use theoretical atomistic models to propose specific changes in protein sequences to improve the interactions between PKs and their substrates. In addition, our theoretical model can be used to analyze the atomistic interactions that have positive or negative influence into the binding affinity of PK-substrate complexes.

The success of this simple protocol also demonstrates that it is not necessary to investigate the activation free energies of the substrate molecules to get information about PK specificities. Those investigations require of sophisticated and complex calculations (such as QM/MM) using a higher level of theory. Instead, MM-GBSA is a popular method because of its high speed and low computational cost.
